# Sex differences in clinical presentation of systemic lupus erythematosus

**DOI:** 10.1186/s13293-019-0274-2

**Published:** 2019-12-16

**Authors:** Jorge I. Ramírez Sepúlveda, Karin Bolin, Johannes Mofors, Dag Leonard, Elisabet Svenungsson, Andreas Jönsen, Christine Bengtsson, the DISSECT consortium, Agneta Zickert, Albin Björk, Anders A. Bengtsson, Andreas Jönsen, Christine Bengtsson, Christopher Sjöwall, Helena Enocsson, Jonas Wetterö, Per Eriksson, Dag Leonard, Elisabet Svenungsson, Guðný Ella Thorlacius, Gunnel Nordmark, Ingrid E. Lundberg, Iva Gunnarsson, Johanna K. Sandling, Jorge I. Ramírez Sepúlveda, Karin Bolin, Kerstin Lindblad-Toh, Lars Rönnblom, Leonid Padyukov, Lina Hultin-Rosenberg, Maija-Leena Eloranta, Marie Wahren-Herlenius, Solbritt Rantapää Dahlqvist, Gunnel Nordmark, Solbritt Rantapää Dahlqvist, Anders A. Bengtsson, Lars Rönnblom, Christopher Sjöwall, Iva Gunnarsson, Marie Wahren-Herlenius

**Affiliations:** 1 Rheumatology Unit, Department of Medicine, Karolinska Institutet, SE-171 76 Stockholm, Sweden; 2 Department of Medical Sciences, Rheumatology and Science for Life Laboratory, Uppsala University, Uppsala, Sweden; 3 Rheumatology, Karolinska University Hospital, Stockholm, Sweden; 4 Department of Clinical Sciences, Rheumatology, Lund University, Skåne University Hospital, Lund, Sweden; 5 Department of Public Health and Clinical Medicine/Rheumatology, Umeå University, Umeå, Sweden; 6 Department of Clinical and Experimental Medicine, Rheumatology/Division of Neuro and Inflammation Sciences, Linköping University, Linköping, Sweden

## Abstract

**Objective:**

Systemic lupus erythematosus (SLE) predominantly affects women, but previous studies suggest that men with SLE present a more severe disease phenotype. In this study, we investigated a large and well-characterized patient group with the aim of identifying sex differences in disease manifestations, with a special focus on renal involvement.

**Methods:**

We studied a Swedish multi-center SLE cohort including 1226 patients (1060 women and 166 men) with a mean follow-up time of 15.8 ± 13.4 years. Demographic data, disease manifestations including ACR criteria, serology and renal histopathology were investigated. Renal outcome and mortality were analyzed in subcohorts.

**Results:**

Female SLE patients presented more often with malar rash (*p* < 0.0001), photosensitivity (*p* < 0.0001), oral ulcers (*p* = 0.01), and arthritis (*p* = 0.007). Male patients on the other hand presented more often with serositis (*p* = 0.0003), renal disorder (*p* < 0.0001), and immunologic disorder (*p* = 0.04) by the ACR definitions. With regard to renal involvement, women were diagnosed with nephritis at an earlier age (*p* = 0.006), while men with SLE had an overall higher risk for progression into end-stage renal disease (ESRD) with a hazard ratio (HR) of 5.1 (95% CI, 2.1–12.5). The mortality rate among men with SLE and nephritis compared with women was HR 1.7 (95% CI, 0.8–3.8).

**Conclusion:**

SLE shows significant sex-specific features, whereby men are affected by a more severe disease with regard to both renal and extra-renal manifestations. Additionally, men are at a higher risk of developing ESRD which may require an increased awareness and monitoring in clinical practice.

## Introduction

Systemic lupus erythematosus (SLE) is an autoimmune disease characterized by multi-organ involvement, dysregulated autoantibody production, and activation of the type I interferon system [1–5]. Among the spectrum of chronic rheumatic diseases, SLE is one of the most overrepresented diseases in women [6], with a female to male ratio of 9–10:1 [7], only surpassed by primary Sjögren’s syndrome (pSS) with a reported ratio of 9–20:1 [8, 9]. Notably, the pre-pubertal and post-menopausal female:male ratios of SLE are considerably lower ranging from 2 to 6:1 and 3–8:1, respectively, compared with those during child-bearing ages [10, 11]. This female preponderance has been widely accepted as a hallmark of SLE and most rheumatic diseases; however, the pathophysiological mechanisms responsible for the sexual dimorphism are still unclear. Many factors have been put forward as an attempt to explain this sex bias: intrinsic sex differences of the immune system [12], sex hormones [13], sex chromosomes [14], sex differences in gene regulation [15], sex-dependent environmental factors [16], and the gut microbiome [17], among others. The interaction and degree of contribution of these factors to the development of an autoimmune disorder is still poorly understood and, thus, an important field of research.

Strikingly, the sex differences in disease susceptibility also resonate at the clinical level, where women and men present distinctive features. Many studies performed in rheumatoid arthritis [18], multiple sclerosis [19], systemic sclerosis [20], and pSS [21, 22] have highlighted sex differences in disease presentation with regard to disease severity, symptoms or comorbidities. For instance, in pSS, men present with more extraglandular manifestations at the time of diagnosis than women [21], and have a higher frequency of lymphoma [22]. Taken together, this body of work suggests that men with rheumatic diseases, despite being less prone to develop them, tend to have a more severe disease phenotype.

In SLE, male sex has also been associated with a more severe form of the disease in terms of clinical manifestations and prognosis, with renal involvement and serological abnormalities such as hypocomplementemia and anti-dsDNA autoantibodies reported as more common in male patients [23]. Additionally, cardiovascular complications are more frequent among men with SLE, contributing to an overall increased organ damage accrual in these patients [24]. Further, male sex has been identified as a risk factor for premature death when diagnosed with SLE [25]. Whether there is a correlation between gender and long-term prognosis in patients with lupus nephritis has not been completely elucidated. While some studies have found male gender to be a risk factor for renal failure [26–29], there is inconsistency across studies, as several studies have not been able to detect such a correlation [30, 31]. This inconsistency could possibly be explained by the retrospective nature of the studies, small sample sizes, bias in referral and selection of the female controls [32]. Delay in diagnosis, health-seeking behavior, and poor treatment compliance in men has been proposed to account for a poorer prognosis in men [32]. Thus, although it is well-known that male sex confers a higher risk for lupus nephritis, there is a need for further studies to clarify whether male patients are also at a higher risk for more severe forms of lupus nephritis and worse outcome.

Hence, in the present study, we aimed at describing sex differences in the clinical presentation of SLE in a large and well-characterized group of patients with a special focus on renal involvement, a potentially severe manifestation observed more frequently among male patients. Further, we aimed at identifying relevant sex differences in the presentation and outcome of renal involvement, including histopathology, progression to end-stage renal disease (ESRD) and mortality.

## Patients and methods

### Patients in the study

The study population consisted of 1226 patients (1060 women and 166 men) of the DISSECT program [22], out of which 1170 fulfilled at least 4 of the 1982 American College of Rheumatology (ACR) classification criteria [33], and the additional 56 cases had a clinical diagnosis of SLE and fulfilled the Fries’ diagnostic principle for SLE [34]. No exclusion criteria were used. Of the patients for whom information on ethnicity was available, 93% were of European descent (908/976), with similar proportions in women (93%, 786/849) and men (96%, 122/127). Mean disease duration from diagnosis to last follow-up for the whole cohort was 15.4 ± 11.4 years; with 15.8 ± 11.6 years for the female group and 13.4 ± 10.2 years for the male group.

The patients were diagnosed and followed at the Departments of Rheumatology at the University Hospitals in Skåne, Linköping, Uppsala and the four most northern counties in Sweden, as well as the Karolinska University Hospital in Stockholm, Sweden. Clinical data with regard to autoantibody status and disease manifestations including ACR criteria items [33], as well as renal histopathology, were retrieved from the patients’ medical records. The study protocol was approved by the regional ethical committee for the respective study center, and the patients gave informed consent.

### Analysis of renal involvement

Data for in-depth analysis of renal involvement was available from a subgroup of the aforementioned SLE cohort. This consisted of 902 patients (780 women and 122 men) from the Departments of Rheumatology at the University Hospitals in Lund, Uppsala, Linköping and Stockholm.

Out of 322 patients with renal involvement, data regarding renal biopsy findings were available for 265 patients (199 female, 66 male). A renal biopsy was conducted in 81% of the female patients (199/247) and 88% of the male patients (66/75), and subsequent biopsies were taken if needed at different time points during the follow-up period. The biopsies were classified according to the World Health Organization (WHO) [35] or the International Society of Nephrology/Renal Pathology Society (ISN-RPS) [36]. In addition, the biopsies were assessed for findings with vascular involvement as observed in anti-phospholipid syndrome-associated nephropathy (APSN) [37], a histological finding characterized by acute thrombotic lesions in glomeruli and/or arterioles (thrombotic microangiopathy) or more chronic vascular lesions in accordance with APSN. In cases with repeated biopsies, the most severe histopathological class is reported.

Further, data regarding progression of renal function impairment was analyzed in a subgroup of patients (the Stockholm cohort). ESRD was defined as reaching a glomerular filtration rate (GFR) of less than 15 mL/min/1.73m^2^ (GFR < 15). Follow-up time was defined as the number of years from nephritis diagnosis to the last follow-up date. Information on time of death was based on patient charts or follow-up in population registers.

### Statistical analysis

For comparison of continuous variables, the Mann-Whitney *U* test was used. The Chi-square test was used when analyzing categorical data, and Fisher’s exact test was employed if the observed frequency of any given cell was < 5 and/or the total number of analyzed individuals was < 40. Data were analyzed with GraphPad Prism 6. Cox proportional hazard modeling was used to estimate hazard ratios (HR) risk for ESRD and death after nephritis diagnosis, comparing males to females. Estimates were adjusted for age and SLE duration at the time of nephritis diagnosis. Data were analyzed using STATA MP 13.0 (StataCorp LP, College Station, TX, USA). In all analyses, *p* values < 0.05 were considered statistically significant.

## Results

### Sex differences in the fulfillment of ACR criteria

The study population consisted of 1226 SLE patients, out of which 87% were female (*n* = 1060) and 13% male (*n* = 166) (*p* < 0.0001, Table 1). Women were diagnosed at an age of 36 ± 15 years (mean, SD), whereas men were diagnosed at 40 ± 19 years of age (mean, SD) (*p* = 0.006). In the cohort, we first analyzed frequencies of the ACR classification criteria [33] items in female and male patients at the inclusion time point and observed significant sex differences in the frequencies of several organ manifestations. Male patients were significantly more often affected by serositis (*p* = 0.0003) (Table 2), both pleuritis and pericarditis (*p* = 0.02 and *p* = 0.004, respectively). Furthermore, fulfillment of the renal disorder criterion was significantly more common in men with SLE (*p* < 0.0001), as reflected by higher frequencies of proteinuria (*p* = 0.001) and cellular casts (*p* = 0.005). Men also presented more often with the immunologic disorder criterion (*p* = 0.04). On the other hand, female patients presented more frequently with malar rash, photosensitivity, oral ulcers and arthritis criteria (*p* < 0.0001, *p* < 0.0001, *p* = 0.01 and *p* = 0.007, respectively) (Table 2). Female and male SLE patients, however, did not differ in the number of fulfilled ACR classification criteria (Table 2).

**Table 1:** Demographic and basic characteristics of the cohort

	Women % (frequency)	Men % (frequency)	*p* value
Sex	87% (1060/1226)	13% (166/1226)	*< 0.0001*
Age at diagnosis (mean ± SD, years)	36 ± 15	40 ± 19	*0.006*
Follow-up time (mean ± SD, years)	15.8 ± 11.6	13.4 ± 10.2	*0.03*
Deceased at last follow-up	10% (105/1060)	16% (27/166)	*0.02*
Age at death (mean ± SD, years)	66.5 ± 15.2	69.8 ± 15.8	0.19

Italisized *p*-values denote significant observations

**Table 2:** Frequencies of fulfilled 1982 ACR criteria

	Women % (frequency)	Men % (frequency)	*p* value
I. Malar rash	55.8% (592/1060)	39.2% (65/166)	*< 0.0001*
II. Discoid rash	24% (255/1060)	18.7% (31/166)	0.13
III. Photosensitivity	66.7% (707/1060)	43.4% (72/166)	*< 0.0001*
IV. Oral ulcer	24.7% (262/1060)	15.7% (26/166)	*0.01*
V. Arthritis	79.2% (840/1060)	69.9% (116/166)	*0.007*
VI. Serositis	41.1% (436/1060)	56% (93/166)	*0.0003*
Pleuritis	36.2% (302/833)	47.1% (57/121)	*0.02*
Pericarditis	16.6% (138/833)	27.3% (33/121)	*0.004*
VII. Renal disorder	29.9% (317/1060)	54.2% (90/166)	*< 0.0001*
Proteinuria	22.5% (88/391)	47.2% (25/53)	*0.0001*
Cellular casts	16.1% (63/390)	32.1% (17/53)	*0.005*
VIII. Neurological disorder	9.1% (97/1060)	11.4% (19/166)	0.35
Seizures	7.2% (61/847)	10.2% (13/127)	0.23
Psychosis	1.7% (14/847)	3.1% (4/127)	0.24
IX. Hematologic disorder	61.5% (652/1060)	60.2% (100/166)	0.76
Hemolytic anemia	7.2% (61/846)	5.5% (7/127)	0.48
Leukopenia	42.7% (362/847)	40.2% (51/127)	0.58
Lymphopenia	35.6% (301/846)	32.3% (41/127)	0.47
Thrombocytopenia	17.9% (152/847)	15.7% (20/127)	0.51
X. Immunologic disorder	65.8% (698/1060)	74.1% (123/166)	*0.04*
Anti-dsDNA	59.5% (504/847)	66.1% (84/127)	0.15
Anti-Sm	14% (118/845)	15% (19/127)	0.76
XI. ANA	98.3% (1042/1060)	98.2% (163/166)	0.92
Number of fulfilled classification criteria (mean ± SD)	5.6 ± 1.5	5.4 ± 1.4	0.18

Italisized *p*-values denote significant observations

### Sex differences in renal involvement and mortality

In 902 patients (122 men/780 women) for which clinical data for in-depth analysis of renal involvement were available, 75/122 (61%) of the men were diagnosed with renal involvement according to the ACR criteria for renal disease [33]. In contrast, only 247/780 (32%) of the women presented with renal involvement (*p* < 0.0001) (Table 3). Histopathological data from kidney biopsies were available for analysis in a subset of cases (*n* = 265/322 cases with renal involvement), and the majority of the cases presented features of lupus nephritis (Table 3). Interestingly, histopathological examination also revealed other types of renal involvement (APSN, vasculitis, IgA nephropathy, tubulointerstitial nephritis or diabetic nephropathy) in a smaller subset of SLE patients. No significant differences in the occurrence of these subtypes were observed between women and men. The histopathological examination revealed that most patients from both sexes had proliferative nephritis (WHO and/or ISN-RPS classification III or IV). In terms of the overall clinical presentation, renal involvement displayed, in some instances, a marked sexual dimorphism. Women were diagnosed with renal involvement at an earlier age (*p* = 0.006), although the timespan from SLE diagnosis to development of renal disease was not significantly different among the sexes (Table 3).

**Table 3:** Sex differences in the presentation of renal involvement^1^

	Women *n* = 780% (frequency)	Men *n* = 122% (frequency)	*p* value
Patients with renal involvement^2^	32% (247/780)	61% (75/122)	*< 0.0001*
Histopathological data available	81% (199/247)	89% (66/75)	0.23
Renal involvement histopathological classification			
Lupus nephritis^3^			
I-II*	14% (28/199)	12% (8/66)	0.84
III-IV**	65% (129/199)	59% (39/66)	0.46
V***	15% (30/199)	20% (13/66)	0.44
APSN^4^	2% (4/199)	3% (2/66)	0.69
Other^5^ histological findings	4% (8/199)	5% (5/66)	0.85
Disease duration to diagnosis of renal involvement^6^ (mean ± SD)	4.8 ± 7.4	4.2 ± 7.1	0.36
Age at diagnosis of renal involvement (mean ± SD)	32.4 ± 14.4	38.8 ± 17.3	*0.006*

^1^Data available for patients followed at the University Hospitals in Linköping, Lund, Stockholm and Uppsala

^2^Diagnoses include lupus nephritis, APS nephropathy, vasculitis, IgA nephropathy, tubulointerstitial nephritis and diabetic nephropathy

^3^According to the World Health Organization (WHO) or International Society of Nephrology/Renal Pathology Society (ISN-RPS) classification. In cases with repeated biopsies, the most severe class was used. Missing histopathological data: female group (48/247) and male group (9/75)

^4^Anti-phospholipid syndrome associated nephropathy (APSN) was defined as APS features present in the renal biopsy

^5^Other histopathological findings, including vasculitis, IgA nephropathy, tubulointerstitial nephritis and diabetic nephropathy

^6^Disease duration to diagnosis of renal involvement = years from SLE diagnosis to onset of renal involvement

*3 females had concomitant findings of APSN/TMA

**3 females and 2 males had concomitant findings of APSN/TMA

***3 males had concomitant findings of APSN/TMA

Italisized *p*-values denote significant observations

Furthermore, we analyzed renal outcome and mortality in a subcohort of patients with histopathologically verified renal involvement from the Karolinska University Hospital (*n* = 166) in which long-term follow-up data were retrieved until date. Importantly, after adjusting for age at diagnosis of renal involvement, analysis by Cox proportional hazard modeling demonstrated that men with SLE had a higher relative risk for development of ESRD, with a hazard ratio of 5.1 (95% CI, 2.1–12.5) (Tables 4 and 5). Further, the Cox modeling also revealed that men with SLE and renal involvement had a trend towards an increased death rate, HR 1.7 (95% CI, 0.8–3.8), in comparison with the corresponding female group.

**Table 4:** Age and disease duration in 166 patients with renal involvement in the Karolinska University Hospital cohort

	Women	Men
*n*	129	37
Age at nephritis diagnosis median, (interquartile range)	31 (24–44)	37 (27–53)
SLE duration at nephritis diagnosis median, (interquartile range)	1 (0–8)	0 (0–2)

**Table 5:** Risk of ESRD and death in men compared with women after diagnosis of renal involvements

Event	No. events		Person-years		Incidence rate per 1000 person-years (95% CI)		Risk estimate (male sex)		Median time to event, years^1^	
	Men	Women	Men	Women	Men	Women	Hazard ratio	95% CI	Men	Women
ESRD	11	15	568	2654	19.3	5.7	5.1	2.1–12.5	14	14
Death	10	33	660	2839	15.2	11.6	1.7	0.8–3.8	8.5	35

^1^In individuals experiencing the event

*ESRD*, end-stage renal disease

## Discussion

The cohort investigated here represents, up to this date, the study with the largest number of male patients ever included in an analysis of clinical sex differences in SLE. The sexual dimorphism in the clinical presentation of SLE has been previously acknowledged [23, 38–41], and based on our present findings, which confirm and extend results from prior publications, it is apparent that women with SLE are significantly more often affected by cutaneous manifestations while men present with a more severe spectrum of organ manifestations.

Renal disorder (proteinuria and/or presence of cellular casts) was observed significantly more often in men with SLE from our cohort, in accordance with previous findings [10, 42]. Lupus nephritis is one of the most severe disease manifestations of SLE; arising from an autoantibody-mediated glomerular inflammation, and dictated in part by a genetic susceptibility [43, 44]. Male SLE patients were not only more prone to present with renal involvement, but they were also more likely to progress into ESRD, a critical complication that can lead to increased mortality [45]. Notably though, the frequency of different histopathological subtypes did not differ between female and male patients. Previous studies have reported that impaired renal function, measured as decreased GFR, was one of the strongest risk factors for mortality in SLE patients [46]. The higher risk of ESRD among men could potentially be explained by other comorbidities such as hypertension, atherosclerosis, tobacco smoking, or hyperlipidemia, which could negatively affect the progression of the renal disease. However, such data were not available for analysis in the current study. We could also demonstrate a clear trend towards an increased mortality in men with renal involvement as compared with women. The lack of firm statistical significance may be explained by limitations in the sample size.

Currently, there are no proposed molecular mechanisms to explain this male propensity to present with renal manifestations. It is of note, though, that men from our cohort had more immunological disturbances. This enhanced humoral response in the male group could exacerbate the inflammation occurring in the renal tissue, contributing to the progression to ESRD observed in our cohort.

Overall, our results suggest a more severe phenotype in male SLE. In contrast to a recent publication [47], the majority of the patients in our study were of European descent, which entails that our findings could represent renal features specific for this population, but not necessarily other populations. In the study by Feldman et al. [47], data were collected from the Medicaid Program, which introduces a selection bias. One strength of the present study is that it includes a large set of unselected SLE patients, since the health care in Sweden ensures that all individuals are seen and diagnosed within the same system. This allows for inclusion in a population-based manner and a possibility for prompt follow-up of patients.

The increased frequency of serositis in male SLE has been recognized in previous studies, where male sex has been identified as a risk factor for the development of pleuritis, but not pericarditis [41, 48–50]. However, in our study, we found that both pleuritis and pericarditis occur more often in men. The male susceptibility for serositis is currently not well understood. Possibly, genetic polymorphisms could partly account for this manifestation. One example of how this may occur is a single nucleotide polymorphism (SNP) in *CXCR3* described by Im et al. [51], which is associated with pleuritis only in male SLE patients. The *CXCR3* gene, situated on the X chromosome, encodes a chemokine receptor which interacts with CXCL9, CXCL10 and CXCL11. The polymorphism may modulate the chemokine axis, promoting a potential increase in lymphocyte migration into target tissues. This process might be enhanced in male SLE patients carrying this SNP on their only X chromosome and, thus, promote inflammation of the pleurae. In general, men with rheumatic diseases present more frequently with pulmonary complications. Rheumatoid pleuritis is more common in male than female patients [52], and men with pSS exhibit interstitial lung disease more frequently than female pSS patients [22]. Thus, it appears that the lung is a specially affected organ in male patients with systemic autoimmunity. Further studies shall aim to clarify the possible pathophysiological mechanisms involved in this sexually dimorphic feature.

On the other hand, several epidemiological studies [53, 54] have described a higher incidence and prevalence of cutaneous lupus erythematosus in women than men. As reported by Jarukitsopa et al. [54], the age-dependent presentation of cutaneous lupus manifestations might hint at a sex hormone-driven process, orchestrated by estrogens. Estrogen may play a crucial role in skin manifestations and flares in SLE and, therefore, have a more negative impact in women due to its higher levels than in men.

This study has several strengths, including the well-characterized SLE population, and the Swedish health care insurance system which offers equal service to all citizens, regardless of socioeconomic or geographic status and thus diminishes inclusion bias. Some limitations should also be mentioned. The participating clinics are tertiary referral centers, suggesting that the included patients may have a more severe disease phenotype than a general SLE study population. A tendency to not diagnose SLE in males may constitute a bias; SLE is known to be unusual among males, and milder skin and joint manifestations in males may potentially pass without specific diagnosis until more specific or obvious manifestations, such as serositis or proteinuria, become apparent.

## Perspectives and significance

Our study highlights and corroborates the notion that male sex is associated with a more severe form of SLE, characterized by an increased propensity for certain phenotypes like serositis and renal disorder. Men with SLE presented more frequently with renal involvement and have a higher risk of progression to ESRD, and there appeared to be a trend towards a higher mortality rate in males with renal involvement. Conversely, women were more often affected by skin manifestations. The identification of these sex differences in SLE manifestations is crucial to raise awareness of a more severe disease course in male patients. This may be of importance in the clinical setting, allowing physicians to increase their surveillance, especially in male lupus patient with renal involvement.

## Data Availability

In concordance with the ethic approval and Swedish law, the data for this study cannot be shared to a third party.
